# The sex specific effect of alcohol consumption on circulating levels of CTRP3

**DOI:** 10.1371/journal.pone.0207011

**Published:** 2018-11-07

**Authors:** Ashley R. DeGroat, Christina K. Fleming, Samantha M. Dunlay, Kendra L. Hagood, Jonathan P. Moorman, Jonathan M. Peterson

**Affiliations:** 1 Department of Biomedical Sciences, East Tennessee State University, Johnson City, Tennessee, United States of America; 2 Department of Health Sciences, College of Public Health, East Tennessee State University, Johnson City, Tennessee, United States of America; 3 Center of Excellence in Inflammation, Infectious Disease and Immunity, James H Quillen College of Medicine, East Tennessee State University, Johnson City, Tennessee, United States of America; 4 Department of Internal Medicine, Division of Infectious, Inflammatory and Immunologic Diseases, Quillen College of Medicine, East Tennessee State University, Johnson City, Tennessee, United States of America; 5 Department of Veterans Affairs, Hepatitis (HCV/HIV) Program, James H Quillen VA Medical Center, Johnson City, Tennessee, United States of America; Oregon Health and Science University, UNITED STATES

## Abstract

The goal of this project was to establish the effect of alcohol consumption on the circulating levels of the adipose tissue derived protein C1q TNF Related Protein 3 (CTRP3). Adipose tissue secretes several adipokines, such as adiponectin and leptin, which exert a multitude of biological effects important for human health. However, adipose tissue is extremely sensitive to alcohol consumption, leading not only to disrupted fat storage, but also to disruptions in adipokine production. Changes to adipokine secretion could have widespread biological effects and potentially contribute to alcohol-induced ailments, such as alcoholic fatty liver disease (ALD). CTRP3 has been previously demonstrated to attenuate fatty liver disease, and suppression of CTRP3 with alcohol consumption could contribute to development of and progression to alcoholic fatty liver disease. To examine the effect of ethanol consumption on circulating adipokine levels, male and female mice were fed an ethanol containing diet (Lieber-DeCarli 5% (v/v) ethanol diet) for 10-days followed by a single gavage of 5 g/kg ethanol (the NIAAA model), or for 6-weeks with no binge added (chronic model). In female mice, adiponectin levels increased ~2-fold in both models of ethanol feeding, but in male mice increased adiponectin levels were only observed after chronic ethanol feeding. On the other hand, in female mice, circulating CTRP3 levels decreased by ~75% and ~50% in the NIAAA and chronic model, respectively, with no changes observed in the male mice in either feeding model. Leptin levels were unchanged with ethanol feeding regardless of model or sex of mice. Lastly, chronic ethanol feeding led to a significant increase in mortality (~50%) in female mice, with no difference in relative ethanol consumption. These findings indicate that ethanol consumption can dysregulate adipokine secretion, but that the effects vary by sex of animal, method of ethanol consumption, and adipokine examined. These findings also indicate that female mice are more sensitive to the chronic effects of ethanol than male mice. Notably, this is the first study to document the effects of ethanol consumption on the circulating levels of CTRP3. Understanding the impact of excessive alcohol consumption on adipokine production and secretion could identify novel mechanisms of alcohol-induced human disease. However, the mechanism responsible for the increased sensitivity remains elusive.

## Introduction

The detrimental effects of chronic alcohol abuse have been well-documented in the setting of established, long-term health conditions such as cardiovascular disease [1], respiratory distress [2], gastrointestinal dysfunction, alcoholic liver disease [3–6], cancer [7], and metabolic dysfunction [8]. Excessive alcohol consumption not only causes initial injury via direct toxic effects (i.e. oxidative stress) to the individual tissues, but also results in secondary indirect injury through elevations in inflammatory cytokines and ectopic fat deposition [9, 10]. Although little can be done to prevent the acute toxic effects of alcohol consumption, understanding and reducing secondary alcohol-induced injury is a key component of treating the long-term health conditions associated with chronic alcohol consumption.

Adipose tissue is not only the primary location for the storage of excess lipids, but is also a major contributor to the production of circulating inflammatory cytokines [11]. Furthermore, chronic alcohol consumption results in high levels of adipose-tissue oxidative stress, leading to elevations in inflammation and hyperlipolysis [11]. Therefore, alcohol-induced disruptions to adipose tissue function, contribute to the wide-spread development of secondary alcohol-related health conditions. Females have a higher amount of adipose tissue (higher percent body fat), and this may account for the increased susceptibility of females to the chronic effects of alcohol [5, 7, 12–14].

In addition to the direct effects of alcohol on adipose tissue, alcohol consumption can also lead to disruptions in adipokine secretion. Adipokines are bioactive proteins secreted by adipose tissue, which can have significant endocrine effects on regulating human health and disease. Circulating levels of the most widely studied adipokines, leptin and adiponectin, are significantly affected by alcohol consumption [10, 15–29]. Leptin is primarily a satiety signal; therefore, its role in ethanol-related health effects is unclear. However, adiponectin stimulates fatty acid oxidation and inhibits both the activity and production of inflammatory cytokines [10, 30, 31], thus supporting our hypothesis that alcohol-induced disruptions to adipokine production can contribute to the development of alcoholic related disease.

In 2004, Wong et al identified a novel family of adipokines, referred to as Complement C1q Tumor Necrosis Factor-Related Proteins (CTRPs) [32, 33]. The initial characterization of these adipose tissue-derived CTRP factors demonstrates wide-ranging effects upon metabolism, inflammation, and survival-signaling [32–52]. As alcohol alters the expression of adiponectin [12, 18, 19, 26–28], we hypothesized that chronic alcohol abuse would also affect other adipokines, specifically CTRP3. We chose to look specifically at CTRP3 because it has previously been shown that overexpressing CTRP3 reduces both high-fat diet induced fatty liver, decreases the synthesis of triglycerides, and decreases circulating levels of the inflammatory cytokine TNF-α [38]. Therefore, the purpose of this project was to establish the effects of alcohol on the circulating levels of CTRP3. The results of this study could produce a new understanding to the mechanism by which chronic alcohol consumption leads to ectopic fat deposition and other health related issues.

## Methods

### Animal model

Forty female mice (C57BL/6) and thirty-seven male mice (C57BL/6) were used for this study. Mice were housed in polycarbonate cages on a 12-h light-dark photocycle with *ad libitum* access to water and food, except as specified. At the time points indicated, animals were anesthetized with isoflurane and euthanized via cardiac puncture. Serum samples were prepared according to manufacturer’s instructions (Sarstedt, Cat#41.1500.005). The gonadal fat pads were excised, snap frozen in liquid nitrogen, and stored at −80°C until further analysis. All animal procedures were conducted in accordance with institutional guidelines, and ethical approval was obtained from the University Committee on Animal Care (protocol #P151201; East Tennessee State University, Animal Welfare Assurance number is A3203-01). Animals were checked/weighed daily and euthanized (counted as dead), via CO_2_ inhalation, based on the presence of any of the following criteria for humane endpoints: unconsciousness, intractable seizures, labored breathing or respiratory distress, inability to ambulate or maintain upright position, diarrhea or constipation, or the inability to eat or drink.

### Ethanol feeding

Two independent ethanol feeding models were employed: The first model was the 11-day chronic plus binge model, also known as the NIAAA model [53]. This model reportedly mimics hepatic steatosis and liver injury, which occurs in many alcoholic hepatitis patients (26). Briefly, 12-week old mice were acclimatized to a control liquid diet (Bio-serv; cat# F1259SP) for 4 days followed by 10 days on the Lieber-DeCarli ethanol diet (5% v/v ethanol; Bio-serv; cat# F1258SP) *ad libitum*. On the morning of the 11^th^ day (1 hour into light cycle) food was removed and replaced with water and the mice were given a single gavage of ethanol (5 g kg^-1^). After gavage, cages were placed on heating pads to prevent hypothermia, as described [54]. Nine hours post gavage, mice were anesthetized with isoflurane, until the absence of reflex was observed, and then euthanized by exsanguination, and tissue/serum samples were collected and processed for analysis.

In the second model (chronic model), 8-week old male and 12-week old female mice were acclimatized to a liquid diet *ad libitum*, without the addition of alcohol for 1-week and then gradually transitioned from 1–5% Lieber-DeCarli ethanol diet (v/v ethanol) over the course of the next 2 weeks, then maintained on 5% ethanol (v/v ethanol) for the remaining 4 weeks. This feeding protocol is believed to reflect chronic ethanol abuse, beginning with low volumes and increasing over time [54]. On the morning of the final day, food was removed, and mice were fasted 9 hours, anesthetized with isoflurane until the absence of reflex was observed, and the euthanized by exsanguination, and tissue/serum samples were collected and processed for analysis. The 9-hour time point was selected to be consistent with the NIAAA model protocol [53].

Control fed mice were placed on an ethanol free isocaloric control diet (Bio-serv; cat# F1259SP) supplemented with maltose dextrin (to match the calories of ethanol), for use as experimental controls. Food intake and body weight were measured daily and mice on the control diet had their food intake limited to match the average total intake for the previous day of the corresponding group of ETOH-fed mice. Mice were group housed 2–4 mice per cage and the food intake was calculated as total/mouse. Food intake and food intake normalized to body weight data were measured and calculated daily for each mouse. The combined average daily values for each mouse were then compared.

### Immunoblot analysis

Serum samples were diluted 1:5 in assay buffer (50 mM Tris HCl, pH 8.0, 150 mM NaCl, 0.1% Triton x-100, 0.5% sodium deoxycholate, 0.1% SDS), plus the addition of protease inhibitors (Bimake Cat#B14001). Afterwards an equal volume of 2x SDS loading buffer (final concentration: 1% SDS, 5% 2-mercaptoethanol, 10% glycerol, 0.004% bromophenol blue, 0.125 M Tris HCl, pH 6.8) was added and the samples were denatured for 5 minutes at 95°C. For each sample 1 μl serum was separated by gel electrophoresis (BioRad; cat#456–1046) and transferred to a nitrocellulose membrane (BioRad; Cat#162–0115), according the manufacturer’s instructions. To confirm appropriate protein migration a protein standard was loaded with each blot (BioRad cat#1610374 or Thermo Scientific cat#26616). Membranes were blocked in 2% non-fat milk and probed with primary antibodies: CTRP3 (R and D Systems Cat# AF2436, RRID:AB_2067713) and Adiponectin (R and D Systems Cat# MAB1119, RRID:AB_2305045). After incubation with primary antibodies membranes were washed and probed with appropriate HRP-labeled secondary antibodies: Rabbit anti-goat (Thermo Fisher Scientific Cat# 31402, RRID:AB_228395) and rabbit anti-rat (Thermo Fisher Scientific Cat# PA1-28786, RRID:AB_10983740). Chemiluminescence signals were visualized with Millipore (Cat# 17010A2). Quantification of signal intensity was performed using Alphaview Software (Alpha Innotech). Due to limited number of wells on a western blot and to avoid blot-to-blot comparisons an n = 6 was used for immunoblot analysis.

### RNA isolation

RNA was isolated according to commercial assay following manufacturer’s instructions (Direct-zol Cat# R2070). Isolated RNA was eluted in 50 μl RNase-free water; purity (RIN ≥ 7.0) and concentrations were confirmed by microfluidic capillary electrophoresis (Agilent RNA 6000 Nano kit, #5067–1511, Agilent Technologies). 1 μg RNA was reverse transcribed according to manufacturer’s instructions (Promega, Cat#A5001).

### Quantitative real-time PCR

A 10-fold dilution series of DNA amplicons generated from a prepared sample was employed as a standard curve for each gene of interest, and the qPCR efficiency was determined for each gene (Bio-Rad Cfx thermocycler). All qRT-PCR primers displayed a coefficient of correlation greater than 0.95 and efficiencies between 90% and 110%. Primer sequences are listed in Table 1. Briefly, 25 ng of cDNA was incubated in SYBR Green qPCR Master mix (Bimake.com, Cat# B21203) for an initial denaturation at 95°C for 10 min, followed by 40 PCR cycles each consisting of 95°C for 15 s, and 60°C for 1 min. After the last cycle specificity of amplification products were confirmed by analyzing melting curve profiles for primers and products. Data is reported as copy number normalized to the geometric mean of the reference genes Beta-actin (Actb) and Hypoxanthine-guanine phosphoribosyltransferase (Hprt1).

**Table 1:** PCR primer sequences.

Gene Name	Forward	Reverse
Actb	CCTCCCTGGAGAAGAGCTATG	TTACGGATGTCAACGTCACAC
Hprt1	CAAACTTTGCTTTCCCTGGT	TCTGGCCTGTATCCAACACTTC
Adipoq	CCTGGCCACTTTCTCCTCATT	ATCCTGAGCCCTTTTGGTGT
CTRP3	CATCTGGTGGCACCTGCTG	TGACACAGGCAAAATGGGAG

Abbreviations: Actb, Beta-actin; Hprt1, Hypoxanthine-guanine phosphoribosyltransferase; Adipoq, Adiponectin; CTRP3, C1q and tumor necrosis factor related protein 3.

### Serum transaminases and pro-inflammatory cytokines

Interleukin-6 (IL-6), Tumor necrosis factor alpha (TNF), Plasminogen activator inhibitor-1 (PAI-1) and leptin were measured using the Bio-Plex Multiplex Immunoassay System according to manufacturer’s instructions (Bio-rad Cat# 171G5023M, 171I50001, 171G5007M). Serum Alanine Aminotransferase (ALT) concentrations were determined using commercially available assays according to manufacturer’s directions (ALT, Fisher Diagnostics Cat#TR71121). Specific assay working ranges, coefficients of variation, and limits of detection are listed in the supplemental data (S1 Table). Any value below the detectable assay working range was assigned the value equivalent to the lower limit of quantification.

### Hepatic lipid analysis

Lipids were extracted as described by Bligh and Dyer and as previously performed [55]. Briefly, liver samples were weighed and homogenized in phosphate-buffered saline (10 ml/g tissue), followed by the addition of 1:2 (vol/vol) chloroform-methanol (3.75 ml/ml of sample homogenate). Next, chloroform was added (1.25 ml/ml of sample homogenate), followed by a final addition of distilled water (1.25 ml/ml of sample homogenate). Samples were vortexed for 30s between each step. Samples were then centrifuged (1,100 g for 10 min at room temperature) to give a two-phase solution (aqueous phase on top and organic phase below). The lower phase was collected with a glass pipette with gentle positive pressure (as to not disturb the upper phase), dried under nitrogen gas at 60°C, and was dissolved in tert-butyl alcohol-Triton X-100 (3:2 vol/vol) solution. Triglycerides were quantified via colorimetric assay according to manufacturer’s directions (Infinity Triglycerides, Fisher Diagnostics, Cat# TR22421).

### Statistical analysis

Descriptive statistics (mean and standard deviations) were calculated for all measured variables. As the NIAAA and chronic feeding models were not performed concurrently, each sex and model were analyzed independently. Final Body weight, average daily food intake, and average daily food intake normalized to daily body weight were analyzed by one-way ANOVA followed by Tukey's multiple comparisons test. All remaining analysis was performed within sex and within diet. Survival curve was determined by log-rank (Mantel-Cox) test. A D'Agostino & Pearson omnibus normality test was used to test for normal distribution in serum analytes. An Unpaired t test was used for normally distributed data and a Mann Whitney U test was used for non-parametric data analysis (TNF-α) to compare data between control and ethanol fed groups within each sex and feeding model for all other variables. All statistical analysis was performed by Graphpad Prism 6, results of ANOVA (F statistics and degrees of freedom) are attached as supplemental data (S1 File).

## Results

### Animal characteristics

No differences in body weight between ethanol and control fed groups were observed. There was a slightly higher food intake for the ethanol fed NIAAA males and the chronic females than their corresponding control fed groups. However, when normalized to body mass, no differences were observed in average daily food intake (ethanol consumption) among ethanol fed and control fed male or female mice (Table 2). Both ethanol feeding protocols demonstrated an increase in hepatic triglycerides and ALT levels. Circulating levels of the transaminase ALT were measured as a marker of hepatic injury and serum cytokines were measured as a marker of systemic inflammation. The cytokine IL-6 was elevated in both feeding models for female mice but was not different in the male mice. The levels of the proinflammatory cytokine TNF-α were below the lower limit of detection in almost all control fed mice. Whereas, in the NIAAA model of ethanol feeding TNF-α was detected in 4/6 females and 6/9 males and in the chronic model of ethanol feeding TNF-α was observed in 9/10 females but only in 3/10 males. Lastly, PAI-1 levels were elevated in both male and female mice regardless of feeding model used. Means, standard deviations and range for all serum values are reported in Table 2.

**Table 2:** Animal characteristics.

	NIAAA model of ethanol feeding			
	Female		Male	
	Con-F	ETOH-F	Con-M	ETOH-M
Animal numbers	6	6	8	9
Final Body weight (g)	18.4 ± 0.6 (17.7, 19.0)	19.7 ± 1.0 (18.5, 21.2)	23.5 ± 1.3# (21.6, 26.0)	24.9 ± 2.6# (20.8, 28.8)
Average daily food intake (g/kg body weight/day)	498 ± 10 (480, 510)	462 ± 8 (450, 469)	429 ± 39# (384, 495)	457 ± 43 (401, 517)
Average daily ethanol intake (g/kg body weight/day)	N/A	17.7 ± 0.52 (16.9, 18.2)	N/A	17.8 ± 2.2 (15.6, 20.3)
Hepatic triglycerides (mg/g tissue)	3.7 ± 0.8 (2.4, 4.6)	7.9 ± 1.6 * (6.1, 10.2)	5.7 ± 2.5 (3.4, 10.3)	10.4 ± 4.3* (4.9, 16.6)
ALT (U/L)	146 ± 71 (56, 274)	360 ± 159 * (145, 599)	79 ± 22 (55, 106)	206 ± 62* (123, 315)
IL-6 (pg/mL)	50.3 ± 40.7 (3.8, 103)	2722 ± 2214* (349, 6097)	168.8 ± 74.2 (65, 226)	1194 ± 1139 (110, 2497)
TNF (pg/mL)	340 ± 157 (241, 592)	610 ± 377 (241, 1123)	264 ± 45 (241, 373)	428 ± 211* (241, 913)
PAI-1 (ng/mL)	3.5 ± 1.2 (1.8, 5.5)	10.2 ± 4.8* (5.4, 15.8)	4.0 ± 1.3 (1.9, 5.1)	9.8 ± 5.0 * (3.6, 15.9)
	Chronic model of ethanol Feeding			
	Female		Male	
	Con-F	ETOH-F	Con-M	ETOH-M
Animal numbers^a^	8	10	6	10
Final Body weight (g)	23.9 ± 2.7 (19.4, 27.8)	25.4 ± 2.9 (22.5, 31.6)	27.5 ± 4.4 (19.4, 33.9)	27.2 ± 2.3 (22.4, 29.9)
Average daily food intake (g/kg body weight/day)	465 ± 31 (405, 496)	484 ± 33 (426, 533)	438 ± 25 (422, 487)	466 ± 63 (367, 608)
Average daily ethanol intake (g/kg body weight/day)	N/A	16.7 ± 0.84 (15.5, 17.5)	N/A	15.4 ± 2.7 (13.3, 19.3)
Hepatic triglycerides (mg/g tissue)	16.2 ± 4.5 (11.2, 22.8)	47.9 ± 11.3* (35.4, 64.8)	26.8 ± 9.5 (17.8, 42.2)	46.0 ± 21.0* (19.1, 80.0)
ALT (U/L)	117± 31 (78, 151)	432 ± 175* (145, 665)	122 ± 26 (79, 145)	182 ± 46* (123, 2630)
IL-6 (pg/mL)	35.7 ± 10 (23.2, 50.2)	79.4 ± 52.7* (29.3, 177)	28.3 ± 13.5 (10.7, 45.8)	44.8 ± 22.9 (20.1, 87.8)
TNF (pg/mL)	330 ± 87 (241, 483)	1292 ± 1657* (241, 6020)	285 ± 68 (241, 373)	513 ± 531 (241, 1641)
PAI-1 (ng/mL)	3.6 ± 0.5 (3.1, 4.5)	7.1 ± 3.0* (3.6, 12.5)	2.7 ± 0.1 (2.5, 2.8)	3.9 ± 0.8* (2.9, 5.2)

Data are reported as: mean ± standard deviation (minimum, maximum). Abbreviations: Con-F, pair-fed female; ETOH-F, ethanol fed female; Con-M, pair-fed male; ETOH-M, ethanol fed male.

^a^Animal number refers to number of mice that completed the study, mice that died during the ethanol feeding were not used for further analysis.

* = p < 0.05 ETOH vs Con.

# = p<0.05 Female vs male.

Abbreviations: Con-F, pair-fed female; ETOH-F, ethanol fed female; Con-M, pair-fed male; ETOH-M, ethanol fed male.

### Animal mortality

**Fig 1:**
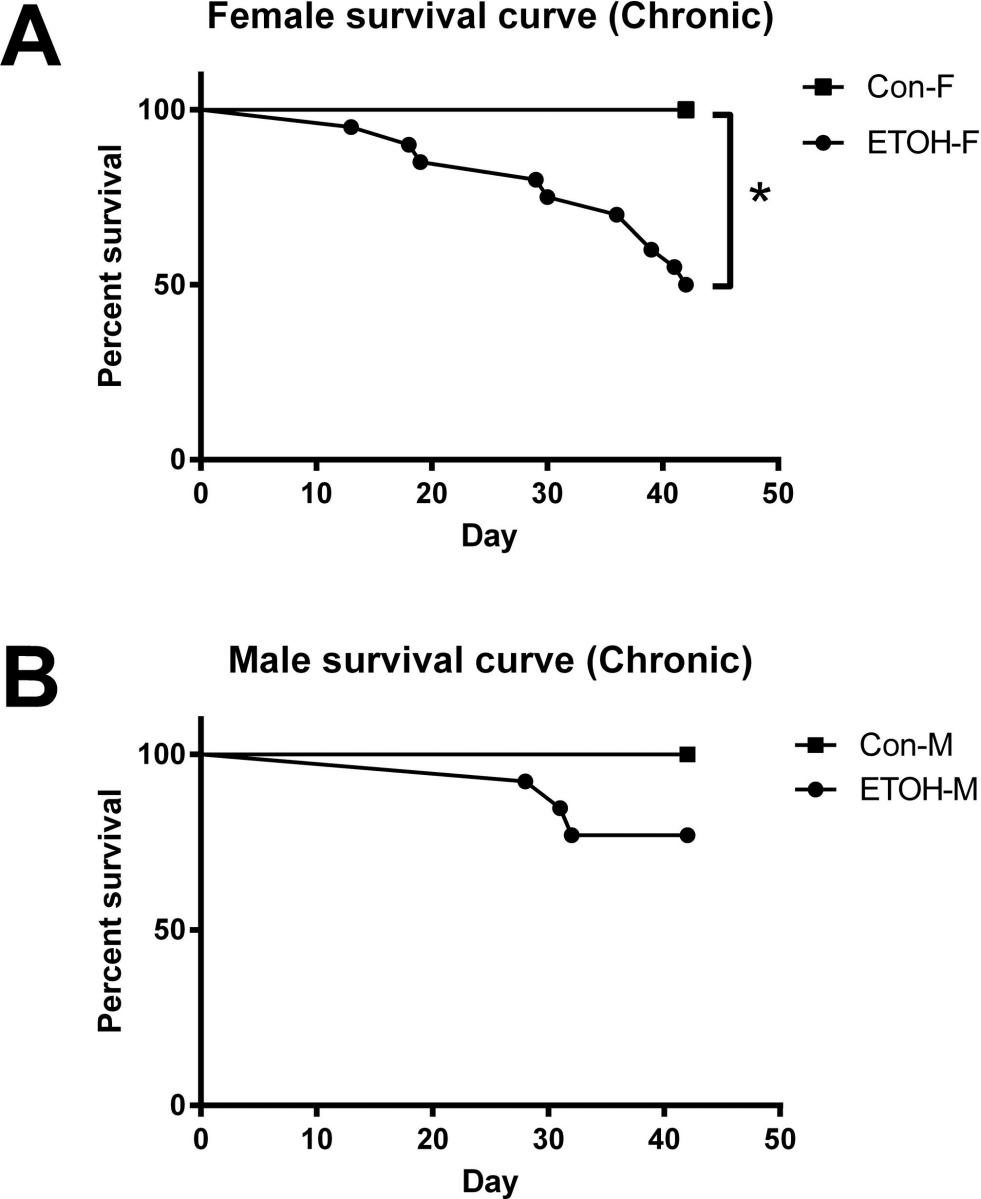
Survival curve with chronic ethanol feeding. The survival curve was plotted for female (A) and male (B) mice on the chronic ethanol feeding model. Data are reported as percent of total, * = p < 0.05 ETOH vs Con. Abbreviations: Con-F, pair-fed female; ETOH-F, ethanol fed female; Con-M, pair-fed male; ETOH-M, ethanol fed male.

All animals on the NIAAA feeding protocol survived through the end of the experiment. Unexpectedly, with 6-weeks of ethanol feeding the female mice suffered 50% mortality compared with a non-significant difference in mortality rate in identically treated male mice (Fig 1). Mice that died prior to completion of the feeding protocol were removed from the study, resulting in a final count of 10 chronic ethanol fed mice for each sex which were used for subsequent analysis.

### Circulating adipokines

**Fig 2:**
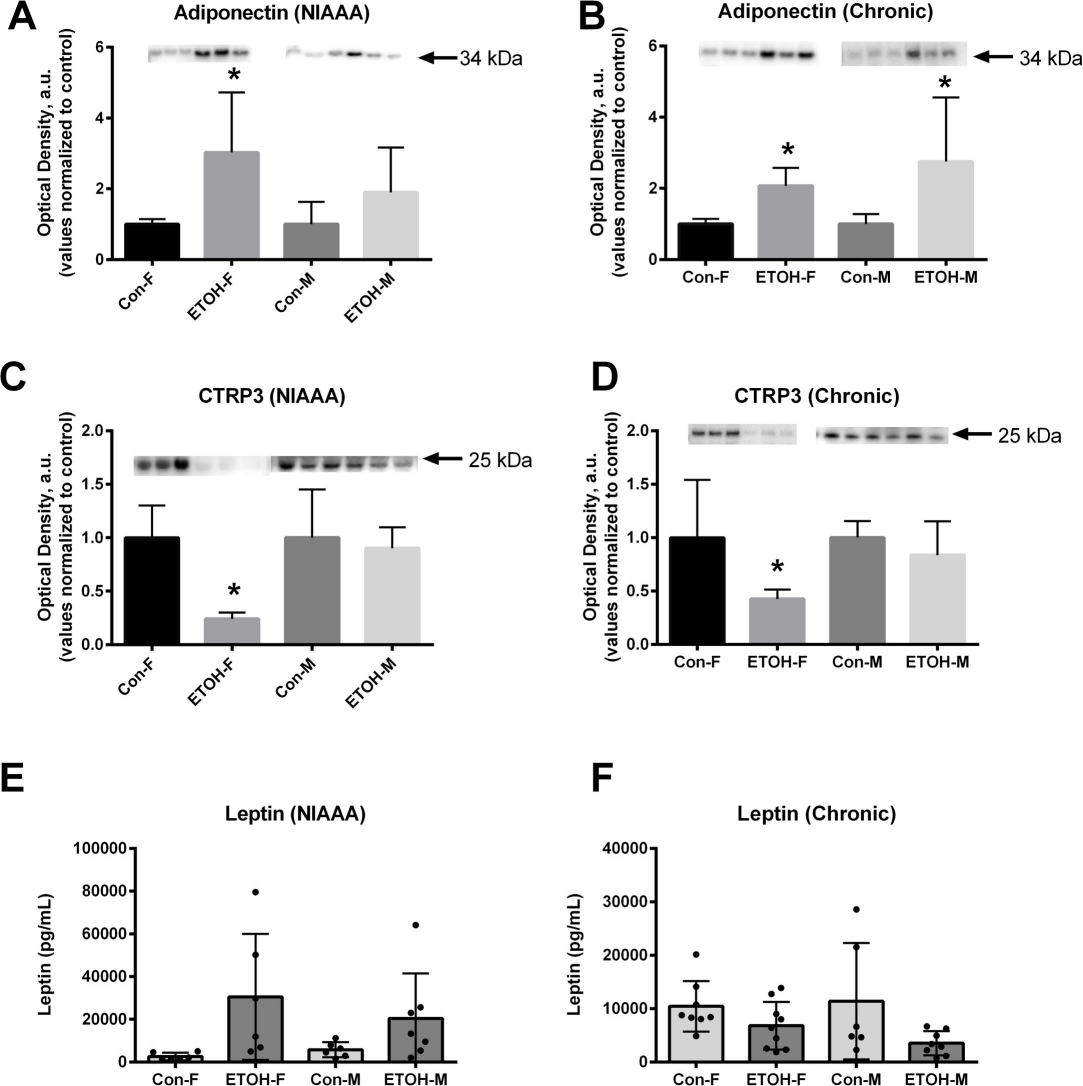
Circulating adipokines in the NIAAA and chronic models of ethanol feeding. Circulating adiponectin and CTRP3 levels were determined by immunoblot in serum collected from ethanol fed mice (n = 6). Circulating leptin levels were determined using multiplex serum analysis. Data are reported as mean ± SD. Male and female blots were performed and analyzed independently, and values were normalized to control fed within each sex. * = p < 0.05 ETOH vs Con. Abbreviations: Con-F, pair-fed female; ETOH-F, ethanol fed female; Con-M, pair-fed male; ETOH-M, ethanol fed male; NIAAA, National Institute on Alcohol Abuse and Alcoholism. Representative immunoblot images are shown, full membrane images are attached as supplemental data (S1 and S2 Figs).

In response to the NIAAA model, female mice fed ethanol had an ~200% increase in circulating levels of adiponectin (Fig 2A) and a ~75% decrease in CTRP3 (Fig 2C). However, ethanol treatment in the male mice led to no change in circulating adiponectin (Fig 2A) or CTRP3 (Fig 2C) levels. With chronic ethanol feeding, circulating adiponectin levels doubled in both the male and female mice (Fig 2B), but again CTRP3 levels were only reduced in the female mice. Interestingly, although leptin levels varied widely, they were not different between control and ethanol fed mice regardless of ethanol feeding model examined (Fig 2E and 2F).

### Adipokine gene expression

**Fig 3:**
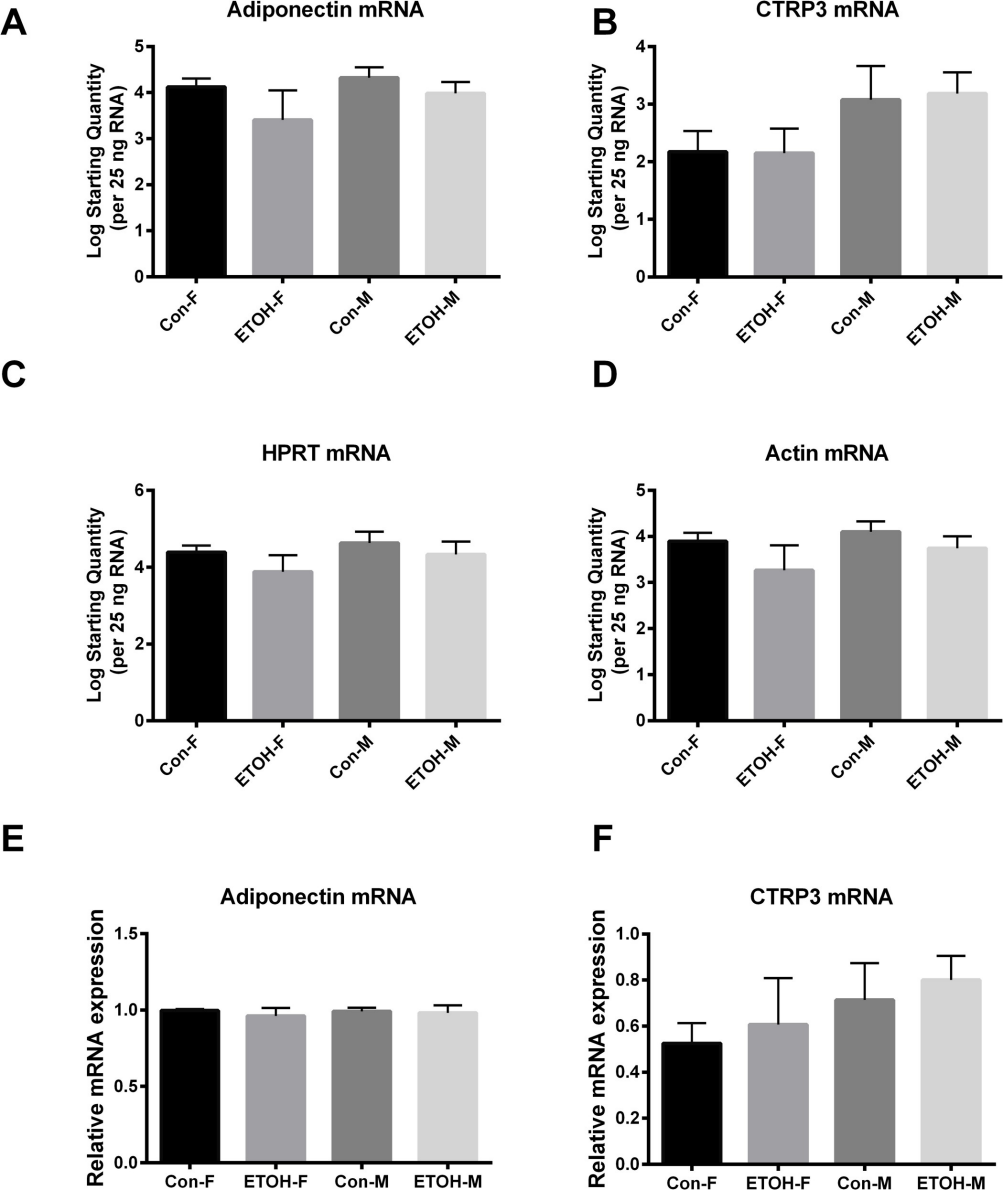
Tissue adipokine gene expression. Gonadal adipokine gene expression was measured in chronically ethanol fed mice. Data are reported as mean of log starting quantity per 25 ng RNA (A-D) or geometric mean ± SD. Abbreviations: Con-F, pair-fed female; ETOH-F, ethanol fed female; Con-M, pair-fed male; ETOH-M, ethanol fed male. Cq values for each sample are shown in supplemental S2 Table.

Gonadal adipose gene expression was analyzed to determine if differences in serum could be contributed to changes in tissue expression. However, no differences were observed in adiponectin or CTRP3 gene expression (Fig 3), indicating that changes to gene expression, at least in the gonadal fat pads, were not responsible for the changes in circulating adipokine levels.

## Discussion

The primary finding of this study is that excessive ethanol consumption effects the concentration of circulating adipokines levels, but not uniformly. Briefly, with alcohol consumption adiponectin levels increased and CTRP3 levels decreased, whereas no significant difference in leptin levels was observed.

The secondary and unanticipated finding of this study is that female mice have a higher mortality rate in response to chronic alcohol feeding male mice. Although increase sensitivity to ethanol feeding has been repeatedly noted in the literature to our knowledge this is the first documentation of mortality with identically treated male and female mice [5, 13, 56]. In this study, male and female mice consumed similar amounts of ethanol, normalized to body weight; however, female mice had significant inflammation (elevations in TNF-α and IL-6) as well as mortality. Conversely, there was no difference in mortality between control and ethanol fed male mice. Interestingly, there was no difference in mortality between ethanol fed male and female mice within the first 4 weeks of ethanol feeding. These data indicate that the female mice are more sensitive to the chronic effects of ethanol consumption. It is important to note that ethanol consumption led to increased levels of PAI-1, indicating in both sexes that alcohol consumption leads to tissue damage and disruptions to overall tissue homeostasis [57].

### Adiponectin

Adiponectin was one the first adipokines discovered and widely studied. Circulating levels of adiponectin have been documented to be affected by alcohol consumption, although to what extent alcohol alters this protein is unclear. In brief, adiponectin levels have been shown to be suppressed [18, 24], not different [26], or elevated [12, 18, 19, 26–28, 58] in response to alcohol consumption. It is suspected that oxidative stress induced by acute alcohol exposure reduces the secretion of adiponectin [29], indicating that time since last dose of ethanol can affect results. As our mice were 9-hrs removed from the last ethanol exposure, any acute suppression of adiponectin secretion that may have occurred would not have been observed. On the other hand, chronic alcohol abuse has demonstrated positive associations between circulating adiponectin levels and the severity of liver damage in cases of cirrhosis [10, 59–61]. Our data supports the finding that circulating adiponectin levels increase with both NIAAA and chronic alcohol consumption [12, 18, 19, 26–28]. Combined, these data indicate that activating adiponectin and adiponectin mediated signaling pathways may not be a successful strategy for the prevention/treatment of alcoholic liver disease, as its levels are already increased with, at least in some models of, chronic ethanol exposure.

### CTRP3

Ethanol feeding reduced circulating CTRP3 levels in female, but not male, mice in response to both the NIAAA and chronic feeding model. Alcoholic cirrhosis occurs at a higher rate in female alcoholic patients, at an earlier age, and with a lower proportional amount of alcohol consumption [5, 12, 13]. As CTRP3 levels are selectively reduced in ethanol fed female mice, this provides a novel mechanism to explore the increased susceptibility of females to alcoholic cirrhosis. Our previous work has shown that CTRP3 acts directly on liver tissue to stimulate lipid oxidation and attenuate both diet-induced and alcoholic fatty liver disease [38, 55]. In support of this, CTRP3 levels are found to be lower in human patients with diet-induced hepatic steatosis, or non-alcoholic fatty liver disease [62]. Further, our lab as well as others have shown that CTRP3 has been shown to prevent inflammation [48, 49, 52, 63, 64]. Chronic alcohol consumption disrupts lipid synthesis which leads to the buildup of hepatic lipids, resulting in alcoholic fatty liver and eventually alcoholic cirrhosis [5, 9, 13], the leading causes of liver failure and a leading cause of death in the Unites States [5, 13, 14, 56]. Thus, there has been renewed interest in developing effective therapeutic strategies to prevent alcoholic fatty liver disease [4, 6, 9, 13, 65, 66]. Our data, combined with the finding that CTRP3 prevents alcohol-induced fatty liver disease [55], identifies CTRP3 as an ideal candidate to develop novel treatments for alcoholic fatty liver disease.

### Sex specific effects of ethanol

For at least 30 years it has been documented that females have an increased morbidity (i.e. alcoholic cirrhosis) with alcohol consumption compared with men, starting at an earlier age and with a lower duration and amount of relative alcohol consumption [67–70]. These findings have been independently confirmed multiple times throughout the literature [5, 12, 13, 56, 67–69, 71–79]. To make matter worse, even after drinking is ceased, females have a worse prognosis than males, indicating sexual dimorphism in the long-term effects of chronic alcohol abuse [68, 69]. Further, in animal experiments with identical feeding protocols there is a significant increase in systemic inflammation, liver inflammation, and hepatic fibrosis in female mice [12, 53, 72, 73, 80], which is in agreement with the results of this study.

Although the mechanisms responsible for the increased sensitivity of females to alcohol remain elusive, there evidence to link these effects to presence of certain female sex hormones. For example, with chronic alcohol consumption endotoxins are released from the gut into the circulation. When animals are exposed to high levels of female sex hormones combined with endotoxins the immune cells generate ~300% more inflammatory cytokines than in response to endotoxins alone [72, 77, 79, 81]. This hyper-activation of the immune system exacerbates any ethanol-induced dysfunction. Interestingly, if the sex hormones are added to isolated macrophages no endotoxin sensitization occurs, indicating a non-direct pathway [72, 77, 79]. CTRP3 is a unique and exciting circulating adipokine as it prevents both endotoxin activation of macrophages [47–49, 52] and alcohol-induced hepatic triglyceride accumulation, at least in male mice [55]. Combined with the data from this manuscript, demonstrating that ethanol feeding leads to a significant reduction in circulating CTRP3 levels in female mice, the ethanol-induced reduction in circulating CTRP3 levels are a potential mechanism for the increased hepatic sensitivity to alcohol in females. However, this premise as well as the effects of sex hormones on circulating CTRP3 levels both require further study.

### Study limitations

All of our data is from a 9-hour fasted time-point, which is sufficient to demonstrate baseline data, but does not demonstrate the metabolic changes to the post-prandial state of these animals. Ethanol ingestion could acutely suppress the secretion of adipokines, such as adiponectin, which could explain the discrepancy among adiponectin levels reported in the literature. Further, although we did not observe significant differences in circulating leptin levels, the unexpectantly high variability combined with our small sample size obscured potential difference between groups. We also only harvested gonadal adipose tissue for RNA analysis, and did not determine adipose tissue weight. It is possible that gene expression differences in other adipose tissue compartments (i.e. subcutaneous) may contribute to changes to circulating adipokine expression or that changes to total amount of adipose tissue could affect circulating CTRP3 levels. Future studies should examine the effects of alcohol on total amount of adipose tissue as well as gene expression at various adipose tissue depots. Lastly, as the animals were group housed, we were unable to account for the exact amount of diet consumed by each individual mouse which could increase the variability in the data analysis. Another source of variability is that because mice were housed 2–4 per cage and died at an uneven rate, the amount of diet (and ethanol) consumed per mouse can only be estimated.

### Conclusions

Since the discovery of leptin and adiponectin, research into understanding the role of adipokines in human health has become a popular topic. This study demonstrates that sex and mode of ethanol exposure can significantly influence the results, indicating that the role of alcohol on adipokines should be studied via multiple models. Lastly, we have identified the sex specific, alcohol-induced reduction in CTRP3 as a potential mechanism for the increased susceptibility of females to alcoholic cirrhosis. These findings warrant further study.

## Supporting information

S1 Fig Supporting data for Fig 2A and 2C, circulating adipokines in the NIAAA model of ethanol feeding. Full membrane of ponceau red stains (A, C, E, G) and the chemiluminescence images (B, D, F, H) are shown for proteins as indicated. The protein standards (Cat# 161–0374 or 26612) have been labeled with corresponding protein molecular weights. All samples on the blot were analyzed, box indicates representative subset shown in figure. ETOH samples are indicated with a bar under the bands of interest and control samples are left unmarked.
(TIF)

S2 Fig Supporting data for Fig 2B and 2D, circulating adipokines in the chronic model of ethanol feeding. Full membrane of ponceau red stains (A, C, E, G) and the chemiluminescence images (B, D, F, H) are shown for proteins as indicated. The protein standards (Cat# 161–0374 or 26612) have been labeled with corresponding protein molecular weights. All samples on the blot were analyzed, box indicates representative subset shown in figure. ETOH samples are indicated with a bar under the bands of interest and control samples are left unmarked.
(TIF)

S3 Fig CTRP3 antibody specificity. To test the specificity of the CTRP3 antibody we used measured supernant from HEK293 cell culture transfected with expression plasmid to express CTRP1, CTRP2, or CTRP3. This test showed the CTRP3 antibody used in the study is specific for detecting CTRP3 protein detecting a band ~25kDa.
(PNG)

S1 Table Assay working ranges. Specific assay working ranges, coefficients of variation, and limits of detection are listed for multiplex and ALT assay.
(DOCX)

S2 Table qPCR raw data. Raw Cq values and geometric means for adiponectin, CTRP3 are reported. In addition Cq values for reference genes HPRT and Actin for each sample are reported.
(DOCX)

S1 File ANOVA results. The results for the ANOVA including the F statistic, degrees of freedom, and p-values for body weight and food intake data. In addition the results of the Tukey Post Hoc analysis are included.
(XLSX)
