# Supplementary figures and images for: The sex specific effect of alcohol consumption on circulating levels of CTRP3
Source: PLoS One. 2018 Nov 7;13(11):e0207011. doi: 10.1371/journal.pone.0207011 (PMC6221322; doi:10.1371/journal.pone.0207011)

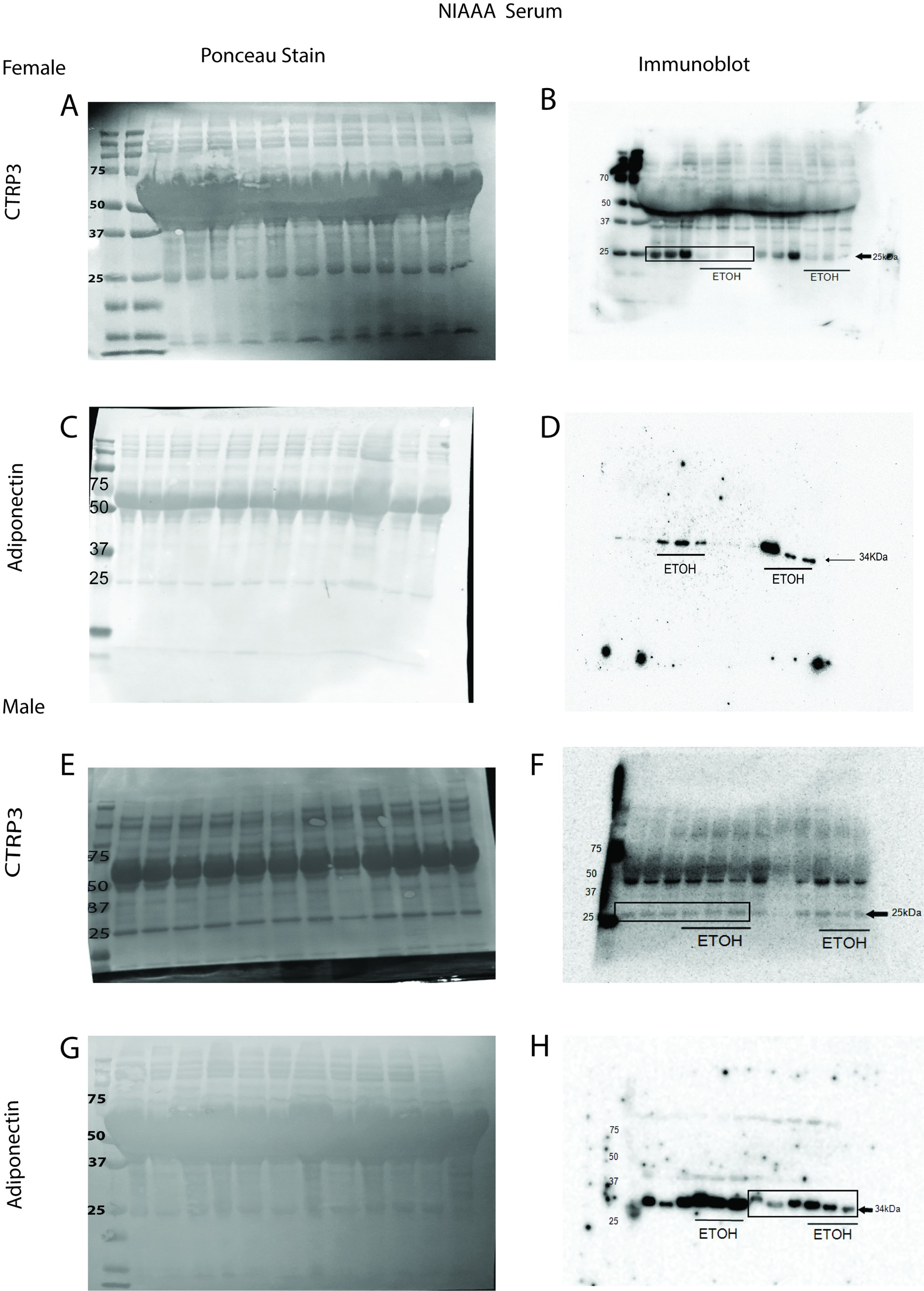

Supplement: S1 Fig — Full membrane of ponceau red stains (A, C, E, G) and the chemiluminescence images (B, D, F, H) are shown for proteins as indicated. The protein standards (Cat# 161–0374 or 26612) have been labeled with corresponding protein molecular weights. All samples on the blot were analyzed, box indicates representative subset shown in figure. ETOH samples are indicated with a bar under the bands of interest and control samples are left unmarked. (TIF) [file pone.0207011.s001.tif]

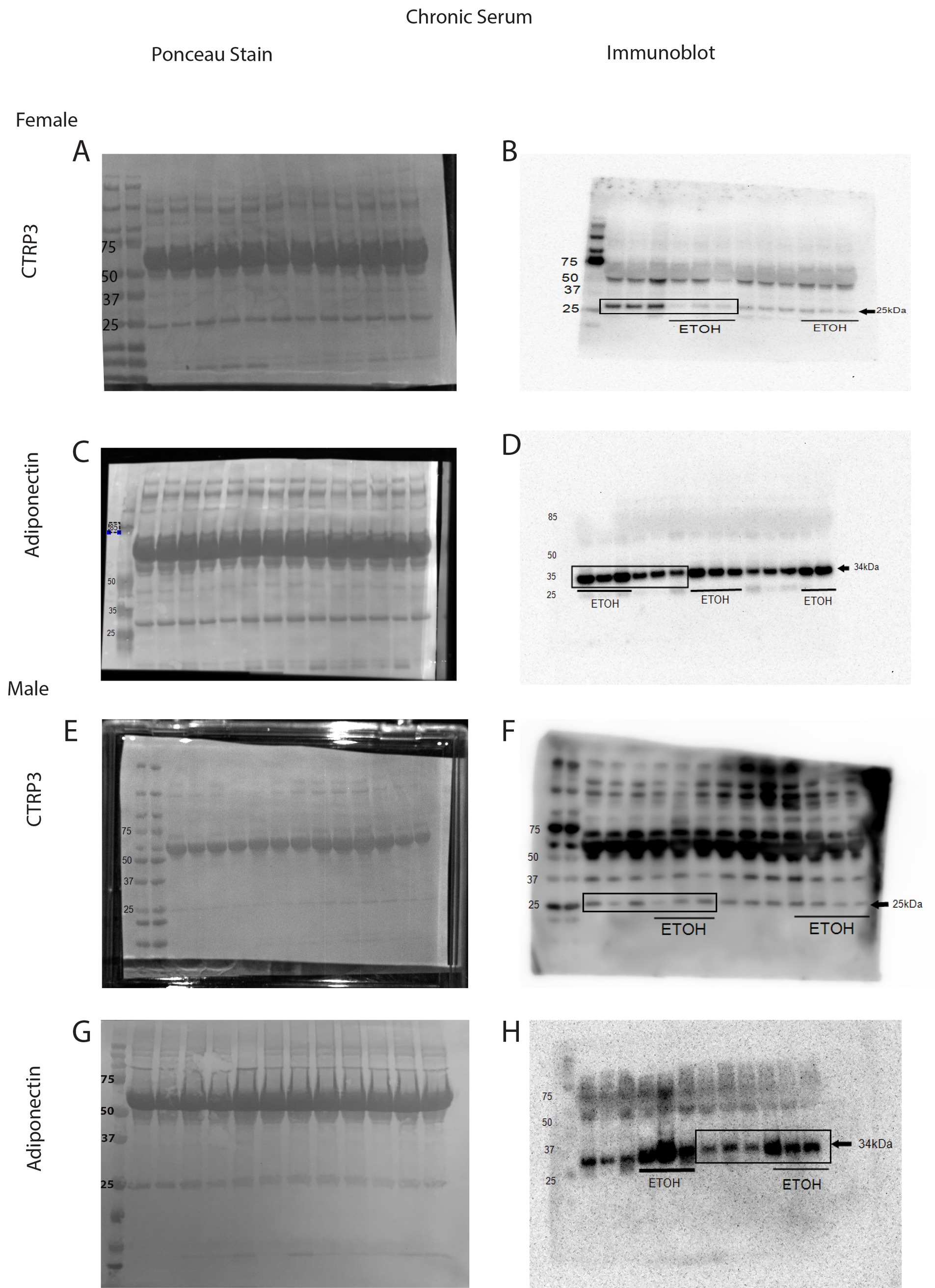

Supplement: S2 Fig — Full membrane of ponceau red stains (A, C, E, G) and the chemiluminescence images (B, D, F, H) are shown for proteins as indicated. The protein standards (Cat# 161–0374 or 26612) have been labeled with corresponding protein molecular weights. All samples on the blot were analyzed, box indicates representative subset shown in figure. ETOH samples are indicated with a bar under the bands of interest and control samples are left unmarked. (TIF) [file pone.0207011.s002.tif]

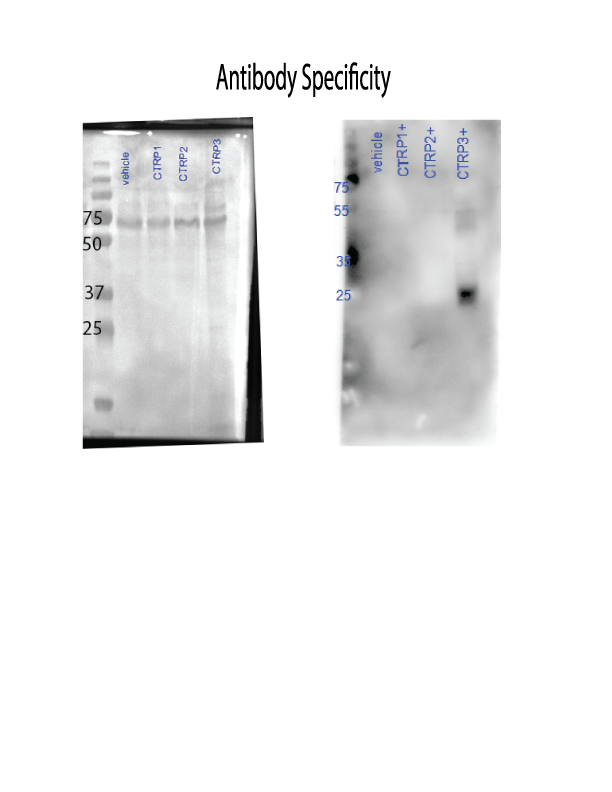

Supplement: S3 Fig — To test the specificity of the CTRP3 antibody we used measured supernant from HEK293 cell culture transfected with expression plasmid to express CTRP1, CTRP2, or CTRP3. This test showed the CTRP3 antibody used in the study is specific for detecting CTRP3 protein detecting a band ~25kDa. (PNG) [file pone.0207011.s003.png]
