# Supplementary material for: The sex specific effect of alcohol consumption on circulating levels of CTRP3
Source: PLoS One. 2018 Nov 7;13(11):e0207011. doi: 10.1371/journal.pone.0207011 (PMC6221322; doi:10.1371/journal.pone.0207011)
Supplement: S1 Table — Specific assay working ranges, coefficients of variation, and limits of detection are listed for multiplex and ALT assay. (DOCX) [file pone.0207011.s004.docx]

Supplementary Table S1

|  | Assay Working Ranges, | | Assay Precision | |
| --- | --- | --- | --- | --- |
|  | Lower Limit of Quantification | Upper Limit of Quantification | Intra-assay (%CV) | Inter-assay (%CV) |
| Leptin (pg/mL) ^#^ | 11.7 | 51,353 | 2.3 | 3 |
| IL-6 (pg/mL) ^#^ | 29 | 14100 | 3.4 | 16 |
| TNF (pg/mL) ^#^ | 261 | 61420 | 2.9 | 6 |
| PAI-1 (pg/mL) ^#^ | 0.8 | 12,073 | 3.3 | 2 |
| ALT (U/L) | 20 | 450 | 6.9 | 10 |

#, Data for Inter-assay provision provided by assay supplier.
