# Supplementary material for: The sex specific effect of alcohol consumption on circulating levels of CTRP3
Source: PLoS One. 2018 Nov 7;13(11):e0207011. doi: 10.1371/journal.pone.0207011 (PMC6221322; doi:10.1371/journal.pone.0207011)
Supplement: S2 Table — Raw Cq values and geometric means for adiponectin, CTRP3 are reported. In addition Cq values for reference genes HPRT and Actin for each sample are reported. (DOCX) [file pone.0207011.s005.docx]

|  |  |  | | |  | Normalized (geoMean) | | |
| --- | --- | --- | --- | --- | --- | --- | --- | --- |
| **Sex** | **Diet** | **CTRP3** | **HPRT** | **Actin** | **Adiponectin** | **Adjusted CTRP3** | **Adjusted Adiponectin** |  |
| Female | Control | 1.9 | 4.69 | 4.09 | 4.39 | 0.433 | 1.004 |  |
| Female | Control | 2.1 | 4.23 | 3.68 | 4.00 | 0.524 | 1.013 |  |
| Female | Control | 2.2 | 4.56 | 4.18 | 4.36 | 0.512 | 0.999 |  |
| Female | Control | 2.4 | 4.51 | 4.02 | 4.25 | 0.556 | 0.998 |  |
| Female | Control | 2.9 | 4.36 | 3.92 | 4.09 | 0.703 | 0.990 |  |
| Female | Control | 1.7 | 4.30 | 3.77 | 3.93 | 0.419 | 0.978 |  |
| Female | Control | 2.0 | 4.20 | 3.73 | 3.96 | 0.501 | 1.001 |  |
| Female | Control | 2.2 | 4.30 | 3.77 | 4.01 | 0.554 | 0.994 |  |
| Female | Ethanol | 1.3 | 4.18 | 3.62 | 3.88 | 0.339 | 0.997 |  |
| Female | Ethanol | 1.6 | 3.85 | 3.37 | 3.38 | 0.431 | 0.940 |  |
| Female | Ethanol | 1.9 | 3.66 | 2.68 | 2.80 | 0.615 | 0.892 |  |
| Female | Ethanol | 1.9 | 4.01 | 3.46 | 3.63 | 0.506 | 0.974 |  |
| Female | Ethanol | 2.4 | 4.29 | 3.82 | 4.04 | 0.602 | 0.997 |  |
| Female | Ethanol | 2.3 | 4.04 | 3.50 | 3.73 | 0.603 | 0.992 |  |
| Female | Ethanol | 2.4 | 3.94 | 3.16 | 3.44 | 0.689 | 0.975 |  |
| Female | Ethanol | 2.0 | 4.01 | 3.22 | 3.63 | 0.566 | 1.010 |  |
| Female | Ethanol | 2.3 | 3.99 | 3.48 | 3.69 | 0.615 | 0.991 |  |
| Female | Ethanol | 2.4 | 2.62 | 1.81 | 1.86 | 1.102 | 0.853 |  |
| Male | Control | 3.7 | 4.58 | 4.11 | 4.46 | 0.857 | 1.028 |  |
| Male | Control | 2.9 | 4.72 | 4.22 | 4.43 | 0.650 | 0.992 |  |
| Male | Control | 2.6 | 5.06 | 4.39 | 4.52 | 0.561 | 0.961 |  |
| Male | Control | 3.7 | 4.26 | 3.80 | 3.97 | 0.912 | 0.986 |  |
| Male | Control | 2.5 | 4.54 | 4.00 | 4.24 | 0.587 | 0.994 |  |
| Male | E | 3.1 | 4.46 | 3.80 | 4.15 | 0.757 | 1.009 |  |
| Male | E | 3.5 | 3.83 | 3.45 | 3.55 | 0.969 | 0.978 |  |
| Male | E | 3.6 | 4.45 | 3.94 | 4.30 | 0.853 | 1.026 |  |
| Male | E | 2.5 | 4.27 | 3.51 | 3.98 | 0.644 | 1.029 |  |
| Male | E | 3.0 | 4.60 | 3.91 | 3.95 | 0.715 | 0.931 |  |
| Male | E | 3.3 | 4.76 | 4.10 | 4.13 | 0.738 | 0.935 |  |
| Male | E | 3.3 | 3.97 | 3.50 | 3.84 | 0.880 | 1.032 |  |
| Male | E | 3.9 | 5.00 | 4.22 | 4.19 | 0.847 | 0.912 |  |
